# Predictors of COVID-19 Information Sources and Their Perceived Accuracy in Nigeria: Online Cross-sectional Study

**DOI:** 10.2196/22273

**Published:** 2021-01-25

**Authors:** Olufemi Erinoso, Kikelomo Ololade Wright, Samuel Anya, Yetunde Kuyinu, Hussein Abdur-Razzaq, Abiodun Adewuya

**Affiliations:** 1 Department of Oral and Maxillofacial Surgery Lagos State University Teaching Hospital Lagos Nigeria; 2 Department of Community Health & Primary Health Care Lagos State University College of Medicine Lagos State University Teaching Hospital Lagos Nigeria; 3 Research Unit Lagos State Ministry of Health Lagos Nigeria; 4 Department of Behavioural Medicine Lagos State University College of Medicine Lagos State University Teaching Hospital Lagos Nigeria

**Keywords:** COVID-19, communication, health information, public health, infodemiology, infodemic, accuracy, cross-sectional, risk, information source, predictor, Nigeria

## Abstract

**Background:**

Effective communication is critical for mitigating the public health risks associated with the COVID-19 pandemic.

**Objective:**

This study assesses the source(s) of COVID-19 information among people in Nigeria, as well as the predictors and the perceived accuracy of information from these sources.

**Methods:**

We conducted an online survey of consenting adults residing in Nigeria between April and May 2020 during the lockdown and first wave of COVID-19. The major sources of information about COVID-19 were distilled from 7 potential sources (family and friends, places of worship, health care providers, internet, workplace, traditional media, and public posters/banners). An open-ended question was asked to explore how respondents determined accuracy of information. Statistical analysis was conducted using STATA 15.0 software (StataCorp Texas) with significance placed at *P*<.05. Approval to conduct this study was obtained from the Lagos State University Teaching Hospital Health Research Ethics Committee.

**Results:**

A total of 719 respondents completed the survey. Most respondents (n=642, 89.3%) obtained COVID-19–related information from the internet. The majority (n=617, 85.8%) considered their source(s) of information to be accurate, and 32.6% (n=234) depended on only 1 out of the 7 potential sources of COVID-19 information. Respondents earning a monthly income between NGN 70,000-120,000 had lower odds of obtaining COVID-19 information from the internet compared to respondents earning less than NGN 20,000 (odds ratio [OR] 0.49, 95% CI 0.24-0.98). In addition, a significant proportion of respondents sought accurate information from recognized health organizations, such as the Nigeria Centre for Disease Control and the World Health Organization.

**Conclusions:**

The internet was the most common source of COVID-19 information, and the population sampled had a relatively high level of perceived accuracy for the COVID-19 information received. Effective communication requires dissemination of information via credible communication channels, as identified from this study. This can be potentially beneficial for risk communication to control the pandemic.

## Introduction

COVID-19, caused by the virus SARS-CoV-2, was first identified in Wuhan, Hubei Province, China, in December 2019 [1], and was thereafter recognized as a pandemic by the World Health Organization (WHO) on March 11, 2020 [2]. As of July 6, 2020, more than 11 million cases and 537,419 deaths related to COVID-19 have been reported in 213 countries [3]. In Africa, over 470,000 cases have been recorded with more than 29,000 cases from Nigeria [3]. The virus is typically spread from one person to another via respiratory droplets and contact with contaminated surfaces [4].

COVID-19 has led to unprecedented local and global public health measures, such as obligatory movement restrictions, social and physical distancing, and prolonged closures of schools and leisure centers. Guidelines on risk factors and preventive measures have emerged in Nigeria from state health agencies, the Nigeria Centre for Disease Control (NCDC), and the Federal Ministry of Health [5]. As our understanding of SARS-CoV-2 increases, local COVID-19 interventions are beginning to focus on the sources and perceived accuracy of disseminated preventive information, which portends an important process in the prevention and control of the disease [6].

Potential sources of information during disease outbreaks include the internet and social media platforms (Facebook, WhatsApp, Instagram, and Twitter), traditional media (television, radio and newspapers), places of worship, health care providers, family members and friends, and workplaces. Therefore, due to the varied sources of information on COVID-19, identifying factors related to such sources can support public educational interventions [7].

In a survey conducted in the Nigerian population during the Ebola virus disease (EVD) outbreak in 2014 [8], the majority of respondents depended on traditional media for information on the disease, while less than one-third depended on the internet for their information. This may suggest that perception and trust of information source are intertwined and contribute to use [8].

As the Nigerian government has begun easing COVID-19 lockdown measures, effective communication is an essential component for mitigating the risks associated with the inevitable clustering of people in public places and other practices capable of not only fueling the spread of the disease but spiking the number of cases and mortality from COVID-19 [9]. Therefore, effectively communicating the efficacy of practical interventions such as personal hygiene, hand washing, use of face masks, and social distancing among other strategies may help curb transmission. Consequently, identifying common sources of COVID-19 information among the population, and the perceived accuracy of these information sources, will possibly guide risk communication processes and the dissemination of evidence-based COVID-19 public health information. The dissemination of this information can be channeled to the most commonly used sources with the highest levels of perceived accuracy.

This study aims to identify the sources of COVID-19 information among Nigerians, as well as the predictors and the perceived accuracy of these sources. Findings from this study will support policymakers in disseminating targeted evidence-based anti–COVID-19 information to populations at risk and those affected. This can invariably empower the public with the capacity to make informed health decisions and improve health outcomes.

## Methods

This study is part of an online survey descriptive cross-sectional survey on COVID-19 conducted by the Lagos State University College of Medicine between April 22 and May 20, 2020. The survey was conducted to assess psychological distress, adherence, and sources of information during the COVID-19 outbreak.

### Study Design and Population

Study participants were consenting individuals aged 18 years or above, residing in Nigeria at the time of the study. A multistage sampling technique was used in selecting study participants. First, 3 states were purposively selected from the 36 states of Nigeria because at the time of study design, they accounted for the highest number of COVID 19 cases in the country [10]. Subsequently, a sample frame of local community networks (estate associations, local organizations, schools, and religious organization groups) within Lagos State, Ogun State, and Federal Capital Territory Abuja was obtained by the Lagos State Ministry of Health (LSMoH) research office using cross-country partners. A list of networks was selected using a simple random sampling method. Leaders of these community networks were identified by the LSMoH research office and contacted via phone and email. Permission was obtained from community network leaders before the online survey link was shared with all members of their respective groups. In total, 9 leaders were approached, and 7 provided consent to share the link to the survey via email and on the social media pages (WhatsApp and Twitter) of their groups. Subsequently, consenting participants on these groups indicated individual consent by clicking the “I consent” button on the first page of the online form before proceeding to answer the survey questions. This data collection approach was used due to the movement restrictions and social/physical distancing measures enforced by the federal government of Nigeria to curb COVID-19 transmission.

### Assessment Tools

Data collection was conducted using an online survey tool, SurveyMonkey (SurveyMonkey Inc). Completing the survey took an average of 7 minutes.

### Study Measures

#### Sociodemographic Variables

Information was obtained on respondents’ sex (male or female), age, marital status, education, and income level (in naira, NGN). Marital status was grouped as single, married, and previously married (ie, widow, widower, divorced, and separated). The educational level of respondents was categorized in three groups: high school or less, university or polytechnic, and postgraduate education. Income level was categorized into three groups based on earnings per month: less than NGN 20,000 (<US $52); NGN 20,000-70,000 (US $52-$181); NGN 70,000-120,000 (US $181-$310); and greater than NGN 120,000 (>US $310).

#### Sources of COVID-19 Information

The study categorized COVID-19 information sources into the following broad groups: (1) family members and friends; (2) places of worship; (3) health care providers (ie, doctors, nurses, pharmacists); (4) the internet, comprising three broad groups: (a) social media (Facebook, WhatsApp, Instagram, and Twitter), (b) news websites, and (c) non–news-related, non–social media websites (eg, blogs, websites of health regulatory organizations such as the WHO and the NCDC); (5) workplace; (6) traditional media (television, radio, or newspapers); and (7) public posters and banners. The total number of sources consulted was determined for each respondent.

#### Perceived Accuracy of COVID-19 Information Source(s)

The perceived accuracy of COVID-19 information was assessed by asking the question “Do you think your source of information is accurate?” to which respondents were given the response options *Yes*, *No*, or *I don’t know*. An open-ended question asking “How do you differentiate between accurate and inaccurate COVID-19 information?” was used to explore how respondents determined the accuracy of COVID-19 information (see survey questions in Multimedia Appendix 1).

### Statistical Analysis

Responses from the survey tool were automatically converted to variables using Microsoft Excel (Microsoft Corp). Demographic data, source(s), perceived accuracy, and the total number of COVID-19 information sources were expressed using descriptive statistics. The association between sociodemographic variables, source(s), perceived accuracy, and the total number of COVID-19 information sources was investigated using bivariate and multivariate logistic regression analysis. Multiple linear regressions were also used to assess the association between the total number of information sources and factors related to COVID-19 information (age, sex, marital status, education, income, and perceived accuracy). *P* values <.05 were considered significant, and tests were two-tailed. Statistical analysis was done using STATA 15.0 software (StataCorp LLC). Qualitative analysis using R (The R Foundation) was used to explore themes and subthemes explaining the determinants of perceived accuracy of COVID-19 information.

### Ethics

Ethics approval was obtained from the Health Research Ethics Committee of the Lagos State University Teaching Hospital (number LREC/06/10/1347).

### Data Sharing

The data sets used and/or analyzed during the study are available from the corresponding author upon reasonable request.

## Results

### Overview

A total of 719 respondents completed the online survey (94.1% response rate); 45 respondents were excluded due to incomplete responses. The mean age of the respondents was 26.9 years (SD 8.8, range 15-69 years). Respondents aged less than 35 years accounted for 88% (n=633), and females made up 54% (n=390) of all study participants. The majority of respondents were single, with a university/polytechnic education and a monthly income of less than NGN 20,000 (Table 1).

**Table 1:** Sociodemographic factors and sources of information.

Variable		Respondents, n (%)
**Age (years)**		
	<35	633 (88.0)
	≥35	86 (12.0)
**Sex**		
	Male	329 (45.8)
	Female	390 (54.2)
**Marital status**		
	Single	571 (79.4)
	Married	141 (19.6)
	Previously married	7 (1.0)
**Education**		
	Secondary school or less	75 (10.4)
	University/polytechnic	516 (71.8)
	Postgraduate	128 (17.8)
**Income (NGN)**		
	<20,000	288 (40.1)
	20,000-70,000	214 (29.8)
	>70,000-120,000	86 (11.9)
	>120,000	131 (18.2)
**Sources of information**		
	Family and friends	269 (37.4)
	Places of worship	86 (12.0)
	Health care providers	210 (29.2)
	Internet	642 (89.3)
	Workplace	90 (12.5)
	Traditional media	452 (62.9)
	Public posters and banners	89 (12.4)
**Total count of information sources**		
	1	234 (32.6)
	2	149 (20.7)
	3	172 (23.9)
	4	88 (12.2)
	5	37 (5.2)
	6	20 (2.8)
	7	19 (2.6)
**Perceived accuracy of information**		
	No	102 (14.2)
	Yes	617 (85.8)

### Sources of COVID-19 Information

Sources of COVID-19 information among respondents ranged from family and friends to public posters and banners. The most common source of information was the internet (n=642, 89.3%), which comprised news via social media handles, websites, blogs, and social media. This was followed by traditional media comprising television, radio, and newspaper/print resources, as reported by 62.9% (n=452) of all respondents. The least common source of information was places of worship (n=86, 12.0%). Out of a total of 7 options in the survey, most respondents used only one source of information (n=234, 32.6%) (Table 1). The sociodemographic characteristics of respondents using each source type is illustrated in Table S1 in Multimedia Appendix 1.

### Family and Friends

More than one-third of respondents (n=269, 37.4%) obtained COVID-19 information from family and friends (Table 1). Bivariate logistic regressions showed that married respondents had lower odds of obtaining COVID-19 information from family and friends compared to single respondents (odds ratio [OR] 0.66, 95% CI 0.44-0.98) (Table S2 in Multimedia Appendix 1). No significant association was obtained between age, sex, education, income status, perceived accuracy, and family and friends as a source of COVID-19 information.

### Places of Religious Worship

Only 12% (n=86) of respondents obtained COVID-19–related information from their respective places of worship (Table 1). There was no significant association between sociodemographic factors (age, sex, marital status, education, and income) and perceived accuracy with place of worship as a source of COVID-19 information (Tables S2 and S3 in Multimedia Appendix 1).

### Health Care Providers

In total, 29% (n=210) of respondents obtained COVID-19 information from their health care providers (Table 1). Married respondents had lower odds of obtaining COVID-19 information from health care providers compared to single respondents (OR 0.60, 95% CI 0.38-0.93). In addition, respondents with a university or postgraduate degree had lower odds of obtaining COVID-19 information from health care providers compared to respondents with a secondary school degree or less (Table S2 in Multimedia Appendix 1). Respondents earning a monthly income of NGN 70,000-120,000 had 1.75 higher odds of obtaining COVID-19 information from health care providers compared to respondents earning less than NGN 20,000 (95% CI 1.05-2.91).

Table S3 in Multimedia Appendix 1 shows multivariate logistic regressions assessing the effect of sociodemographic factors on information source. Being married and having a university/polytechnic education was significantly associated with reduced odds compared to being single and with a secondary school education or less, respectively. In addition, respondents with a monthly income above NGN 70,000 had significantly higher odds of obtaining COVID-19 information from health care providers compared to respondents earning less than NGN 20,000 (adjusted OR [aOR] 2.21, 95% CI 1.28-3.80).

### Internet

Most respondents (n=642, 89.3%) obtained COVID-19 information from the internet (Table 1). Respondents earning a monthly income between NGN 70,000-120,000 had lower odds of obtaining COVID-19 information from the internet compared to respondents earning less than NGN 20,000 (OR 0.49, 95% CI 0.24-0.98) (Table S2 in Multimedia Appendix 1). However, after adjusting for sociodemographic factors (age, sex, marital status, education) and perceived accuracy, there was no significant association between income levels and use of the internet as a source of COVID-19 information (aOR 0.55, 95% CI 0.26-1.16) (Table S3 in Multimedia Appendix 1).

### Workplace

COVID-19 information from one’s workplace was significantly associated with age, marital status, education, and monthly income. For every 1-year increase in age, there was a 1.04 increase in the odds of obtaining COVID-19 information from the workplace (95% CI 1.02-1.06). Similarly, married respondents had 1.91 higher odds of obtaining COVID-19 information from the workplace compared to single respondents (95% CI 1.16-3.13). Postgraduate education (OR 6.72, 95% CI 1.97-22.96) and higher income (>NGN 120,000; OR 16.78, 95% CI 7.89-35.68) were also significantly associated with the workplace as a source of COVID-19 information (Table S2 in Multimedia Appendix 1). Similarly, in the multivariate logistic regression model, income remained significantly associated with the workplace as a source of COVID-19 information. Respondents earning NGN 20,000 and above had significantly higher odds of obtaining COVID-19 information from their workplace compared to respondents earning less than NGN 20,000 (aOR 3.01, 95% CI 1.32-6.90) (Table S3 in Multimedia Appendix 1).

### Traditional Media

About 62.9% (n=452) of respondents used traditional media as a source of COVID-19 information (Table 1). Older age was significantly associated with the use of traditional media as a source of COVID-19 information (OR 1.02, 95% CI 1.00-1.04). Married respondents also had 1.80 higher odds of using traditional media as a source of COVID-19 information (OR 1.80, 95% CI 1.20-2.71) compared to single respondents (Table S2 in Multimedia Appendix 1). In a multivariate logistic regression analysis, after adjusting for age, sex, education, income, and perceived accuracy, being married remained significantly associated with using traditional media as a source of COVID-19 information (aOR 1.83, 95% CI 1.04-3.25) (Table S3 in Multimedia Appendix 1).

### Posters and Banners

About 12% (n=89) of the respondents surveyed reported that they obtained COVID-19 information from public posters and banners. Female respondents had 0.56 lower odds of using this source of information compared to males (95% CI 0.36-0.88). On the other hand, respondents with a postgraduate education had 2.75 higher odds of obtaining COVID-19 information from public posters and banners compared to respondents with a secondary school education or less (95% CI 0.99-7.63). In the multivariate logistic regression analysis, the use of public posters for obtaining COVID-19 information remained significantly associated with lower odds in females compared to male (aOR 0.55, 95% CI 0.34-0.88). Additionally, respondents with postgraduate education still had higher odds of obtaining information from posters and banners compared to secondary school or less (aOR 4.24, 95% CI 1.36-13.19) (Table S3 in Multimedia Appendix 1).

### Association Between COVID-19 Information Sources and Perceived Accuracy

Table 2 shows a multiple linear regression model demonstrating that participants who earn greater than NGN 120,000 used 0.45 more information sources on average (β=.45, 95% CI 0.07-0.83), compared to those who earn less than NGN 20,000 per month. Therefore, respondents who earn more are more likely to seek information from various sources. Moreover, we devised a Poisson regression model with sources of information as the dependent variable, and sociodemographic factors and perception of accuracy as independent variables (Table S4, Multimedia Appendix 1). Compared to respondents earning less than NGN 20,000, the difference in the log count of the number of information sources increased by 0.16 for respondents earning NGN 120,000 or more, while holding age, sex, marital status, educational level, and perceived accuracy constant (95% CI 0.02-0.32). No significant association was seen between age, sex, marital status, education, and the number of COVID-19 information sources.

**Table 2:** Association between sociodemographic factors and the number of information sources using a multiple linear regression analysis.

Variable		β (95% CI)	*P* value
Age (years)		–0.00 (–0.02 to 0.02)	.99
**Sex**			
	Male	1	
	Female	–0.11 (–0.33 to 0.13)	.37
**Marital status**			
	Single	1	
	Married	–0.30 (–0.70 to 0.09)	.14
	Previously married	–0.32 (–1.48 to 0.83)	.58
**Education**			
	Secondary school or less	1	
	University/polytechnic	–0.10 (–0.47 to 0.28)	.62
	Postgraduate	0.73 (–0.42 to 0.57)	.77
**Income (NGN)**			
	<20,000	1	
	20,000-70,000	0.08 (–0.19 to 0.36)	.56
	>70,000-120,000	0.19 (–0.19 to 0.57)	.33
	>120,000	0.45 (0.07 to 0.83)	.02^a^
**Perceived accuracy of information**			
	Inaccurate	1	
	Accurate	0.06 (–0.26 to 0.38)	.73

^a^*P*<.05.

The majority (n=617, 85.8%) of respondents reported that their source(s) of information was accurate (Table 1). However, there was no statistically significant association between perceived accuracy and each source of information or the number of sources (Tables S2 and S3 in Multimedia Appendix 1; Table 2).

One major theme emerged by assessing respondents’ means of differentiating between accurate and inaccurate COVID-19 information. Respondents (n=129) used information from recognized local and international health regulatory organizations (ie, reputable sources), such as the NCDC, the COVID-19 Presidential Task Force in Nigeria (PTF), the LSMoH, the WHO, and reference to a government or official agencies to determine accuracy. Some open-ended responses in this cohort of respondents include:

I rely on confirmed media accounts of Government agencies NCDC, LSMOH, COVID19 PTF.

56-year-old married male

I cross check with the verified source of information e.g. WHO, NCDC, LSMOH.

25-year-old single female

Among respondents who perceived their source(s) of information as accurate, 83 were confident of the accuracy of the information from the NCDC:

Any news different from the NCDC's is not always accurate.

27-year-old married female

In addition, for some respondents (n=38), information from the WHO website was deemed accurate. This could be inferred from statements like:

When it's from a verified source, like WHO, I know it's accurate.

25-year-old single male

Other ways of determining accuracy include cross-checking multiple sources (n=37):

When same news repeats itself in different places I see it as accurate.

36-year-old married female

In a subgroup analysis on the use of reputable sources of information, respondents earning between NGN 20,000-70,000 had 2.03 higher odds of using reputable sources compared to respondents earning less than NGN 20,000 (95% CI 1.21-3.38) (Table S5, Multimedia Appendix 1). In addition, Table S6 (Multimedia Appendix 1) distinguishes between the sociodemographic characteristics of respondents who used reputable sources of COVID-19 information and those who used other sources.

## Discussion

### Principal Findings

This study assessed common sources of COVID-19 information for a Nigerian study population during the early stages of the pandemic. Findings from this study suggest that most respondents used the internet as a source of COVID-19 information. Further, more than two-thirds of respondents considered their source(s) of information as accurate, and one-third depended on only 1 out of the 7 potential sources of COVID-19 information. However, there was no significant association between any of the potential sources of COVID-19 information and perceived accuracy. In addition, high-income earners (>NGN 120,000) had a greater likelihood of using more than one source of COVID-19 information.

The findings have shown that the internet was the most common source of information among the respondents. This contrasts with findings from the EVD outbreak in Nigeria 6 years ago, where traditional media (television and radio) were the main sources of EVD information at that time [11]. Similarly, a study in Vietnam listed mass media and peer educators as the most common sources of COVID-19 information as opposed to the internet [12]. Nonetheless, several studies in Nigeria and Asia have identified social media (a component of the internet information source) as an important source of information, particularly serving as the first source of information during the 2014 EVD outbreak in Nigeria [7,11-13]. Given the worldwide advancement in technology, the internet may be a common source of COVID-19 information now more than before because of the increased access to smartphones and internet networks in the country [14-17]. Aside from greater access to the internet, access to real-time health information, with audio-visual tools such as YouTube, which can enhance user attention, and respondent demographics (ie, young, educated, and living in major cities) could have contributed to increased internet use during the present pandemic in this study [16,17]. Despite this, there is broad consensus that misinformation is highly prevalent on the internet, and challenges such as limited reach to underserved populations may caution against overreliance on the internet for health communication [18]. Nonetheless, using the internet—through social media handles and websites—for public health messaging accomplishes several of the goals of successful health communication. These goals include reaching a broad audience, creating interactive and ongoing community engagement, and broadening the transmission of urgent public health information [6,14].

Of note in this study is the relatively low proportion of respondents who depended on health care providers for COVID-19–related information. This was particularly common in more educated respondents (university education and above) compared to those with secondary school education or below. On the other hand, respondents with a higher income were more likely to obtain COVID-19 information from health care providers compared to the lowest category of earners in the population studied. A possible explanation for this finding could be that higher-income earners are more likely to have access to health care providers compared to low-income earners through health insurance or their own ability to pay out of pocket. In addition, higher-income earners are more likely to be able to afford medical consultations, facilitating direct access to COVID-19 information from health care providers. Similarly, higher-income earners were more likely to obtain COVID-19 information from their workplace. This finding could be ascribed to a likelihood of having more stable white- or blue-collar jobs compared to lower-income earners who are mostly students on stipends, small business entrepreneurs, and daily wage earners.

Perceived accuracy was not significantly associated with any particular source of COVID-19 information; however, respondents determined the accuracy of their COVID-19 information by cross-referencing with perceived reputable sources of information (ie, internet sources run by national or multinational health regulatory organizations), such as the NCDC and the WHO digital media handles. In an analysis of sources of information, high-income earners had more than 2 times higher odds of using reputable sources of information compared to low-income earners. Overall, our findings correspond with the literature, which indicates that a large proportion of the population relies on the media, as well as family and friends, to inform their perception of health risks during an outbreak [6].

### Limitations

Limitations in the interpretation of findings from this study could be the mode and language of data collection. Since the study used an online survey in the English language, respondents were individuals with access to the internet who could communicate in English, alienating segments of the population with limited access to the internet or without English language fluency. Nonetheless, it is worth noting that individuals with internet access can serve as sources of information for those with limited or no access to the internet. In addition, respondents were predominantly youth, with university/polytechnic education, and earning less than NGN 20,000 per month, therefore limiting the generalizability of our findings.

### Conclusion

The internet was the most common source of COVID-19 information, and the population sampled had a relatively high level of perceived accuracy for the COVID-19 information received. Further, high-income earners had a higher likelihood of using multiple sources of COVID-19 information. To determine the accuracy of COVID-19 information, a significant fraction of respondents cross-referenced any information received with news from official government regulatory bodies (eg, the NCDC) and the WHO.

The dissemination of timely and accurate health information to support public health interventions is crucial during a pandemic. Therefore, targeted and evidence-based approaches must be implemented for effective communication. Findings from this study can inform health communication measures to mitigate the effects of the COVID-19 pandemic on the population and reduce the burden and spread of the disease.

Effective communication requires sensitivity to social perceptions and dissemination of information via relevant communication channels [19,20], as identified from this study. Policymakers responsible for COVID-19 risk communication in Nigeria may consider measures such as an increased focus on the internet (eg, use of NCDC social media handles and local traditional media stations with an online presence). In addition, white- and blue-collar employers can be encouraged to promote anti–COVID-19 health behaviors by conducting health education exercises for employees using official digital channels. Future studies should examine COVID-19 content across various potential sources to better understand the information obtained by the public.

## Appendix

Multimedia Appendix 1 Supplemental materials.

## Abbreviations

aOR: adjusted odds ratio
EVD: Ebola virus disease
LSMoH: Lagos State Ministry of Health
NCDC: Nigeria Centre for Disease Control
OR: odds ratio
PTF: COVID-19 Presidential Task Force
WHO: World Health Organization
