# Supplementary material for: Predictors of COVID-19 Information Sources and Their Perceived Accuracy in Nigeria: Online Cross-sectional Study
Source: JMIR Public Health Surveill. 2021 Jan 25;7(1):e22273. doi: 10.2196/22273 (PMC7837450; doi:10.2196/22273)
Supplement: Multimedia Appendix 1 [file publichealth_v7i1e22273_app1.docx]

Supplementary files

SURVEY QUESTIONS

**A.1** Age [at last birthday]: **--------- (**in years**)**

**A.2** Sex: a. Female ( ) b. Male ( )

**A.3** Marital status: [select only one option]

a. Single []

b. Married []

c. Divorced []

d. Widow/ Widower []

e. Separated []

**A.4** Educational level [select only one option]

a. High school or Less than high school []

b. College/ Polytechnic []

c. Postgraduate []

**A.5** What is your monthly income? [select only one option]

a. <NGN20,000 []

b. NGN20,000-70,000 []

c. NGN70,000-120,000 []

d. NGN>120,000 [].

**A.6** Where do you obtain your information about COVID-19 from? [select all that apply]

1. Family members or friends []
2. Place of worship []
3. Health Care providers (Doctors/Nurses/Pharmacists) []
4. Internet (Facebook/WhatsApp/Instagram/twitter/websites/ blogs) []
5. Work-place []
6. Traditional media (television or Radio or Newspapers) []
7. Public posters and banners []
8. Others (please specify……………………………...)

**A.7** Do you think your source of information is accurate? [select only one option]

a. Yes []

b. No []

**A.8** How do you differentiate between accurate and inaccurate COVID-19 information? [open ended response] ………………………………………………………………………………………………………………

Table S1. Descriptive statistics of demographic variables and perceived accuracy by COVID-19 Information sources

|  | **Variables** | **Family and Friends**  **n (%)** | **Place of worship**  **n (%)** | **Health care provider**  **n (%)** | **Internet**  **n (%)** | **Work-place**  **n (%)** | **Traditional media**  **n (%)** | **Public posters and banners**  **n (%)** |
| --- | --- | --- | --- | --- | --- | --- | --- | --- |
|  |  | 269 (100) | 86 (100) | 210 (100) | 642 (100) | 90 (100) | 452 (100) | 89 (100) |
| Age [In years] | <35 | 243 (90.3) | 75 (87.2) | 192 (91.4) | 567 (88.3) | 71 (78.9) | 387 (85.6) | 82 (92.1) |
|  | ≥35 | 26 (9.7) | 11 (12.8) | 18 (8.6) | 75 (11.7) | 19 (21.1) | 65 (14.4) | 7 (7.9) |
| Sex | Male | 112 (41.6) | 41 (47.7) | 104 (49.5) | 297 (46.3) | 49 (54.4) | 215 (47.6) | 52 (58.4) |
|  | Female | 157 (58.4) | 45 (52.3) | 106 (50.5) | 345 (53.7) | 41 (45.6) | 237 (52.4) | 37 (41.6) |
| Marital Status | Single | 224 (83.3) | 70 (81.4) | 178 (84.8) | 517 (80.5) | 63 (70.0) | 343 (75.9) | 74 (83.1) |
|  | Married | 42 (15.6) | 16 (18.6) | 30 (14.3) | 120 (18.7) | 27 (30.0) | 103 (22.8) | 15 (16.9) |
|  | *Previously married | 3 (1.1) | 0 (0) | 2 (0.9) | 5 (0.8) | 0 (0) | 6 (1.3) | 0 (0) |
| Educational | ≤High school | 32 (11.9) | 8 (9.3) | 33 (15.7) | 69 (10.7) | 3 (3.3) | 45 (10.0) | 5 (5.6) |
|  | University | 195 (72.5) | 60 (69.8) | 141 (67.1) | 459 (71.5) | 59 (65.6) | 318 (70.3) | 63 (70.8) |
|  | Postgraduate | 42 (15.6) | 18 (20.9) | 36 (17.1) | 114 (17.8) | 28 (31.1) | 89 (19.7) | 21 (23.6) |
| Income | <NGN 20,000 | 114 (42.4) | 32 (37.2) | 73 (34.8) | 263 (41.0) | 9 (10.0) | 180 (39.8) | 32 (36.0) |
|  | NGN 20,000-70,000 | 83 (30.9) | 26 (30.2) | 66 (31.4) | 189 (29.4) | 18 (20.0) | 133 (29.4) | 27 (30.3) |
|  | NGN70,000-120,000 | 31 (11.5) | 7 (8.1) | 32 (15.2) | 72 (11.2) | 17 (18.9) | 53 (11.7) | 10 (11.2) |
|  | >NGN 120,0000 | 41 (15.2) | 21 (24.4) | 39 (18.6) | 118 (18.4) | 46 (51.1) | 86 (19.0) | 20 (22.5) |

* Previously married (divorced/ separated/ widow/ widower) divorced/ separated

Table S2. Sociodemographic factors related to sources of COVID-19 information.

| Variables | Family and friends | | Place of worship | | Health care provider | | Internet | | Workplace | | Traditional media | | Public posters and banners | |
| --- | --- | --- | --- | --- | --- | --- | --- | --- | --- | --- | --- | --- | --- | --- |
|  | OR (95% CI) | *P* value | OR (95% CI) | *P* value | OR (95% CI) | *P* value | OR (95% CI) | *P* value | OR (95% CI) | *P* value | OR (95% CI) | *P* value | OR (95% CI) | *P* value |
| Age | 0.98 (0.96,1.00) | 0.06 | 1.01 (0.99,1.03) | 0.43 | 0.99 (0.97,1.01) | 0.19 | 0.99 (0.96,1.01) | 0.33 | 1.04 (1.02,1.06) | 0.00* | 1.02 (1.00,1.04) | 0.03* | 1.0 (0.97,1.02) | 0.80 |
| Sex |  |  |  |  |  |  |  |  |  |  |  |  |  |  |
| Male | 1 |  | 1 |  | 1 |  | 1 |  | 1 |  | 1 |  | 1 |  |
| Female | 1.31 (0.96,1.77) | 0.09 | 0.92 (0.58,1.44) | 0.71 | 0.81 (0.59,1.11) | 0.19 | 0.83 (0.51,1.33) | 0.43 | 0.67 (0.43,1.05) | 0.08 | 0.82 (0.61,1.11) | 0.21 | 0.56 (0.36,0.88) | 0.01* |
| ^a^ Marital Status |  |  |  |  |  |  |  |  |  |  |  |  |  |  |
| Single | 1 |  | 1 |  | 1 |  | 1 |  | 1 |  | 1 |  | 1 |  |
| Married | 0.66 (0.44,0.98) | 0.04* | 0.92 (0.51,1.63) | 0.77 | 0.60 (0.38,0.93) | 0.02* | 0.60 (0.35,1.03) | 0.06 | 1.91 (1.16,3.13) | 0.01* | 1.80 (1.20,2.71) | 0.01* | 0.80 (0.44,1.44) | 0.46 |
| Educational |  |  |  |  |  |  |  |  |  |  |  |  |  |  |
| ≤High school | 1 |  | 1 |  | 1 |  | 1 |  | 1 |  | 1 |  | 1 |  |
| University | 0.82 (0.50,1.33) | 0.42 | 1.10 (0.50,2.40) | 0.81 | 0.48 (0.29,0.79) | 0.00* | 0.70 (0.29,1.69) | 0.43 | 3.10 (0.95,10.14) | 0.06 | 1.07 (0.65,1.76) | 0.79 | 1.95 (0.76,5.01) | 0.17 |
| Postgraduate | 0.66 (0.36,1.18) | 0.16 | 1.37 (0.56,3.33) | 0.49 | 0.50 (0.27,0.90) | 0.02* | 0.71 (0.26,1.93) | 0.5 | 6.72 (1.97,22.96) | 0.00* | 1.52 (0.84,2.76) | 0.17 | 2.75 (0.99,7.63) | 0.05 |
| Income |  |  |  |  |  |  |  |  |  |  |  |  |  |  |
| <NGN 20,000 | 1 |  | 1 |  | 1 |  | 1 |  | 1 |  | 1 |  | 1 |  |
| NGN 20,000-70,000 | 0.97 (0.67,1.39) | 0.86 | 1.11 (0.64,1.92) | 0.72 | 1.31 (0.89,1.95) | 0.17 | 0.72 (0.40,1.29) | 0.27 | 2.85 (1.25,6.47) | 0.01* | 0.99 (0.68,1.42) | 0.94 | 1.16 (0.67,1.99) | 0.61 |
| NGN 70,000-120,000 | 0.86 (0.52,1.42) | 0.56 | 0.71 (0.30,1.67) | 0.43 | 1.75 (1.05,2.91) | 0.03* | 0.49 (0.24,0.98) | 0.04* | 7.64 (3.26,17.87) | 0.00* | 0.96 (0.59,1.58) | 0.88 | 1.05 (0.49,2.24) | 0.89 |
| >NGN 120,0000 | 0.70 (0.45,1.08) | 0.10 | 1.53 (0.85,2.77) | 0.16 | 1.25 (0.79,1.98) | 0.34 | 0.86 (0.43,1.75) | 0.68 | 16.78 (7.89,35.68) | 0.00* | 1.15 (0.74,1.77) | 0.54 | 1.44 (0.79,2.63) | 0.23 |
| **Accuracy |  |  |  |  |  |  |  |  |  |  |  |  |  |  |
| Inaccurate | 1 |  | 1 |  | 1 |  | 1 |  | 1 |  | 1 |  | 1 |  |
| Accurate | 0.76 (0.49,1.16) | 0.20 | 1.02 (0.53,1.96) | 0.95 | 1.59 (0.96,2.63) | 0.07 | 0.89 (0.44,1.80) | 0.75 | 1.21 (0.62,2.37) | 0.57 | 1.28 (0.84,1.96) | 0.26 | 0.72 (0.40,1.30) | 0.28 |

**P*<.05. **Accuracy: for the purpose of this study synonymous with Perceived accuracy of information.

^a^ Previously married was excluded because it had only 7 respondents.

Table S3. Multiple logistic regression analysis of factors related to sources of COVID-19 information.

| Variables | Family and friends | | Place of worship | | Health care provider | | Internet | | Workplace | | Traditional media | | Public posters and banners | |
| --- | --- | --- | --- | --- | --- | --- | --- | --- | --- | --- | --- | --- | --- | --- |
|  | aOR (95% CI) | *P* value | aOR (95% CI) | *P* value | aOR (95% CI) | *P* value | aOR (95% CI) | *P* value | aOR (95% CI) | *P* value | aOR (95% CI) | *P* value | aOR (95% CI) | *P* value |
| Age | 1.00 (0.97,1.02) | 0.84 | 1.02 (0.98,1.06) | 0.34 | 0.99 (0.96,1.02) | 0.67 | 1.00 (0.97,1.04) | 0.80 | 1.00 (0.97,1.04) | 0.83 | 1.00 (0.98,1.03) | 0.76 | 0.98 (0.94,1.02) | 0.35 |
| Sex |  |  |  |  |  |  |  |  |  |  |  |  |  |  |
| Male | 1 |  | 1 |  | 1 |  | 1 |  | 1 |  | 1 |  | 1 |  |
| Female | 1.29 (0.94,1.78) | 0.11 | 1.02 (0.64,1.62) | 0.94 | 0.84 (0.60,1.18) | 0.31 | 0.88 (0.53,1.45) | 0.61 | 0.94 (0.57,1.54) | 0.81 | 0.79 (0.58,1.09) | 0.15 | 0.55 (0.34,0.88) | 0.01* |
| Marital Status |  |  |  |  |  |  |  |  |  |  |  |  |  |  |
| Single | 1 |  | 1 |  | 1 |  | 1 |  | 1 |  | 1 |  | 1 |  |
| Married | 0.71 (0.41,1.24) | 0.23 | 0.55 (0.23,1.31) | 0.18 | 0.53 (0.28,0.98) | 0.04* | 0.51 (0.24,1.09) | 0.08 | 0.59 (0.28,1.25) | 0.17 | 1.83 (1.04,3.25) | 0.04* | 0.68 (0.29,1.56) | 0.36 |
| Educational |  |  |  |  |  |  |  |  |  |  |  |  |  |  |
| ≤High school | 1 |  | 1 |  | 1 |  | 1 |  | 1 |  | 1 |  | 1 |  |
| University | 0.84 (0.51,1.39) | 0.50 | 1.10 (0.50,2.44) | 0.81 | 0.48 (0.29,0.80) | 0.01* | 0.74 (0.30,1.82) | 0.51 | 2.13 (0.62,7.30) | 0.23 | 1.05 (0.63,1.74) | 0.87 | 2.30 (0.88,6.03) | 0.09 |
| Postgraduate | 0.85 (0.43,1.67) | 0.64 | 1.28 (0.46,3.53) | 0.63 | 0.55 (0.27,1.10) | 0.09 | 0.99 (0.32, 3.04) | 0.98 | 2.06 (0.53,8.01) | 0.30 | 1.23 (0.62,2.44) | 0.56 | 4.24 (1.36,13.19) | 0.01* |
| Income |  |  |  |  |  |  |  |  |  |  |  |  |  |  |
| <NGN 20,000 | 1 |  | 1 |  | 1 |  | 1 |  | 1 |  | 1 |  | 1 |  |
| NGN 20,000-70,000 | 0.98 (0.67,1.42) | 0.90 | 1.07 (0.61,1.88) | 0.80 | 1.28 (0.85,1.92) | 0.24 | 0.69 (0.38,1.26) | 0.23 | 3.01 (1.32,6.90) | 0.01* | 0.95 (0.65,1.38) | 0.79 | 1.22 (0.70,2.14) | 0.49 |
| NGN 70,000-120,000 | 0.96 (0.57,1.62) | 0.88 | 0.70 (0.29,1.70) | 0.43 | 2.21 (1.28,3.80) | 0.00* | 0.55 (0.26,1.16) | 0.12 | 8.30 (3.47,19.82) | 0.00* | 0.77 (0.46,1.30) | 0.33 | 1.10 (0.50,2.44) | 0.81 |
| >NGN 120,0000 | 0.88 (0.52,1.50) | 0.65 | 0.48 (0.71,3.06) | 0.30 | 1.81 (1.04,3.16) | 0.04* | 0.98 (0.42,2.26) | 0.96 | 19.79 (8.50,46.09) | 0.00* | 0.75 (0.44,1.26) | 0.28 | 1.32 (0.63,2.77) | 0.46 |
| Accuracy |  |  |  |  |  |  |  |  |  |  |  |  |  |  |
| Inaccurate | 1 |  | 1 |  | 1 |  | 1 |  | 1 |  | 1 |  | 1 |  |
| Accurate | 0.72 (0.47,1.11) | 0.13 | 1.04 (0.54,2.00) | 0.91 | 1.56 (0.93,2.59) | 0.09 | 0.87 (0.43,1.76) | 0.69 | 1.45 (0.71,2.98) | 0.31 | 1.35 (0.88,2.09) | 0.17 | 0.70 (0.39,1.28) | 0.25 |

* *P*<.05.

**Accuracy: for the purpose of this study synonymous with Perceived accuracy of information.

Table S4. Association between sociodemographic factors and number of ^1^information sources using a Poisson regression model.

| Variable | β (95% CI) | p value |
| --- | --- | --- |
| Age [in years] | 0.00 (-0.01,0.01) | 0.99 |
| Sex |  |  |
| Male | 1 |  |
| Female | -0.04 (-0.14,0.05) | 0.39 |
| Marital status |  |  |
| Single |  |  |
| Married | -0.12 (-0.28,0.05) | 0.16 |
| Previously married | -0.13 (-0.63,0.37) | 0.60 |
| Education |  |  |
| Secondary school or less | 1 |  |
| University/ Polytechnic | -0.04 (-0.19,0.12) | 0.63 |
| Postgraduate | 0.03 (-0.17,0.23) | 0.80 |
| Income [in NGN] |  |  |
| <20,000 | 1 |  |
| 20,000-70,000 | 0.03 (-0.08,0.15) | 0.58 |
| >70,000-120,000 | 0.07 (-0.08,0.23) | 0.35 |
| >120,000 | 0.16 (0.02,0.32) | 0.03* |
| Perceived accuracy of information |  |  |
| Inaccurate | 1 |  |
| Accurate | 0.02 (-0.11,0.16) | 0.74 |

* p value <0.05. ^1^ a range of 1-7 sources of COVID-19 information sources.

Table S5. Association between the use of a ‘Reputable’ source of COVID-19 information and sociodemographic factors

|  | ^1^Use of a ‘*Reputable*’ COVID-19 information source |  |
| --- | --- | --- |
| Variable | aOR (95% CI) | p value |
| Age [in years] | 0.98 (0.95,1.01) | 0.18 |
| Sex |  |  |
| Male | 1 |  |
| Female | 1.09 (0.72,1.65) | 0.68 |
| Education |  |  |
| Secondary school or less | 1 |  |
| University/ Polytechnic | 0.67 (0.36,1.26) | 0.21 |
| Postgraduate | 0.79 (0.35,1.82) | 0.59 |
| Income [in NGN] |  |  |
| <20,000 | 1 |  |
| 20,000-70,000 | 2.03 (1.21,3.38) | 0.01* |
| >70,000-120,000 | 2.33 (1.18,4.59) | 0.01* |
| >120,000 | 2.95 (1.51,5.75) | 0.001* |
| Perceived accuracy of information |  |  |
| Inaccurate | 1 |  |
| Accurate | 1.98 (0.99,3.95) | 0.054 |

^1^‘Reputable’ information source was determined using a response to Q.A.8 [see survey questions] indicating use of any of the following as a source of information for differentiating between accurate and inaccurate COVID-19 information: NCDC, WHO, LSMOH, FMOH, CDC, COVID-19 PTF, and official government websites or social media handles. * p value <0.05.

Table S6. Descriptive analysis of Population who used ‘reputable’ information sources

| Study characteristic |  | Reputable source (%) | Other source (%) | p value |
| --- | --- | --- | --- | --- |
|  |  | n = 118 (100) | n = 601 (100) |  |
| Age | <35 years | 99 (83.9) | 534 (88.2) |  |
|  | ≥ 35 years | 19 (16.1) | 67 (11.2) |  |
| Sex | Male | 53 (44.9) | 276 (45.9) | 0.84 |
|  | Female | 65 (55.1) | 325 (54.1) |  |
| Marital status | Single | 96 (81.4) | 475 (79.0) | 0.47 |
|  | Married | 22 (18.6) | 119 (19.8) |  |
|  | ^1^Previously married | 0 (0) | 7 (1.2) |  |
| Education | Secondary school or less | 16 (13.6) | 59 (9.8) | 0.22 |
|  | University/ Polytechnic | 77 (65.2) | 439 (73.0) |  |
|  | Postgraduate | 25 (21.2) | 103 (17.2) |  |
| Income [in NGN] | <20,000 | 31 (26.3) | 257 (42.8) | 0.01* |
|  | 20,000-70,000 | 42 (35.6) | 172 (28.6) |  |
|  | >70,000-120,000 | 17 (14.4) | 69 (11.5) |  |
|  | >120,000 | 28 (23.7) | 103 (17.1) |  |
| Perceived accuracy | Inaccurate | 10 (8.5) | 92 (15.3) | 0.052 |
|  | Accurate | 108 (91.5) | 509 (84.7) |  |

* p value <0.05.^1^ Previously married: Divorced, separated, widow, widower.
